# Mitigating combined cadmium and microplastics toxicity in rice through nano-zinc modified biochar

**DOI:** 10.3389/fpls.2026.1755367

**Published:** 2026-03-30

**Authors:** Shao Jinhua, Huang Wen’er, Gan Fu, Abdulmohsen I. Algefare, Bilal Ahamad Paray

**Affiliations:** 1 Guangxi Hydraulic Research Institute, Guangxi Key Laboratory of Water Engineering Materials and Structures, Nanning, China; 2 Department of Biological Science, Faculty of Science, King Faisal University, Al-Ahsa, Saudi Arabia; 3 Department of Zoology, College of Science, King Saud University, Riyadh, Saudi Arabia

**Keywords:** biochar modification, gene expression, microplastics, nano-zinc, rice, yield

## Abstract

**Background:**

Cadmium (Cd) and microplastics (MPs) are gradually increasing in soils, posing a serious threat to humans and crop production. Biochar is a important amendment used worldwide for the remediation of contaminated soils. The role of biochar in mitigating combined Cd and MPs toxicity is rarely studied. Thus, we studied the impact of nano-zinc modified biochar (NZMB) on rice growth, functioning, and productivity in Cd and MPs-co-contaminated soil.

**Methods:**

The study has different treatments: control, Cd contaminated soil (20 mg kg^-1^), MPs contaminated soil (1%), Cd + MPs contaminated soil, NZMB (2%), Cd contaminated soil (20 mg kg^-1^) + NZMB (2%), MPs contaminated soil (1%) + NZMB (2%) and Cd + MPs contaminated soil + NZMB (2%).

**Results:**

It was observed that Cd and MPs reduced rice yield (-81%) by impairing chlorophyll synthesis, leaf water contents (-91%), soil nutrient availability, and increasing Cd availability. Biochar application increased the antioxidants activities, osmolyte synthesis, soil organic carbon (+26%), soil pH (18%), nitrogen (+61%), phosphorus (+50%) and potassium availability (40%) and reduced soil Cd availability (-31%), roots Cd (-52%) and shoot Cd (-28%), led to increase in yield in Cd + MPs contaminated soil. Further, NZMB also enhanced the gene expression related to proline (*OsP5CS*), sucrose (*OsSPS1*), and antioxidants, while decreased expression of gene associated with Cd uptake (*OsNRAMP1* and *OsHMA3*), all of which contributed to an increase in rice yield.

**Conclusion:**

This study highlights that NZMB can mitigate the combined Cd and MPs toxicity by decreasing Cd uptake and improving plant functioning. Therefore, these findings will help to develop eco-friendly measures for remediating multi-contaminated soils.

## Introduction

The contamination of agricultural lands and food with toxic metals is a huge challenge to crop production and humans (Rashid et al., 2023). The global population is continuously increasing, and food demands are expected to increase by 35% to 56% between 2010 and 2050 (Van Dijk et al., 2021). Recently, pollution caused by different contaminants, including toxic metals and microplastics (MPs), has posed a serious challenge (Revell et al., 2021). Microplastics have gained widespread attention owing to their serious ecological risks (Wang et al., 2022). They make their entry into soil via plastic films, sewage water, manure, atmospheric sedimentation, and degraded plastic products (Surendran et al., 2023). Microplastics inhibit plant growth (Yu et al., 2020), alters the soil physio-chemical properties, which in turn negatively affect plant growth and development (Dong et al., 2024). Terrestrial plants play a crucial role in the fate of MPs, and they are potential sinks of MPs during their atmospheric transportation (Liu et al., 2020). Cuticle and stomatal absorption and exoplast pathway are important ways of MPs entry into plants (Wang et al., 2023). Thus, they impair plant functioning, cause oxidative damage, decrease nutrients and water absorption and damage the photosynthetic apparatus (Jiang et al., 2024). Notably, MPs enter the human body via contaminated food and cause lung, heart, and nervous system diseases (Prata et al., 2020; Jiang et al., 2024). Thus, it calls for developing the appropriate measures to prevent their entry into soils and mitigate the hazardous impacts of MPs on plants.

Soil contamination with toxic metals is also another serious challenge nowadays. Cadmium (Cd) is a toxic metal with a half-life ranging from 10 to 30 years (Yang et al., 2022). Plants rapidly absorb Cd, which impairs plant function and disturbs minerals uptake, thus causing growth and yield losses (Huang et al., 2023). It also inhibits root growth, carbon fixation, and stomata density (Hassan et al., 2024) and stomata conductance, and leaf water contents (Chen et al., 2018). Additionally, Cd contaminated foods ingested by humans cause heart diseases, cancer and kidney failures (Cirovic and Satarug, 2024). Microplastics affects the migrations of toxic metals in soil-plant systems, thereby imposing positive and negative impacts. For instance, MPs (1%) in Cd-polluted soil, augmented the soil pH and active Cd concentrations (Wang et al., 2020). Other researchers witnessed that MPs (2%) increased Cd availability to plants (Erdem et al., 2025).

Moreover, some researchers found that MPs decrease Cd uptake and its subsequent accretion in plants; nevertheless, it depends on size, the rate of MPs, environmental conditions, and soil properties (Zhang et al., 2022). The combination of Cd and MPs alters microbial diversity and composition and natively impacts carbon and nitrogen cycling, thus decreasing plant growth (Pang et al., 2023). Furthermore, co-exposure of Cd and MPs also inhibits chlorophyll synthesis, alters antioxidant activities, increases reactive oxygen species production, thereby seriously reducing plant growth (Jiang et al., 2024).

Biochar remediates Cd-polluted soils and improve the plant performance (Joseph et al., 2021), because of its higher surface area and presence of excellent functional groups on its surface (Albert et al., 2021). Biochar increases antioxidant activities, photosynthetic efficiency, nutrient uptake, and decreases oxidative damage and Cd uptake (Gul et al., 2024). Recently, biochar modification through different minerals has shown effective results in enhancing stress resilience (Sultan et al., 2025a, b). Zinc improves growth, and provide protection to plants from stress via increasing enzyme activity, osmolyte production, and nutrient uptake (Faran et al., 2019). It is also reduces uptake of Cd, thus decreases oxidative damages and ensure better growth (Zhou et al., 2019). Nano-materials are widely used globally, and they have shown tremendous results in mitigating abiotic stress (Eljamal et al., 2024). Likewise, zinc nanoparticles have shown appreciable results in remediating Cd-polluted soils and mitigating their toxic impacts on plants (Hassan et al., 2024). Biochar can be modified with nano-materials through impregnation and ultrasonic agitation methods (Tan et al., 2020; Chellapandi et al., 2024). The nano-particles enriched biochar reduces Cd availability, thereby increasing root growth, nutrient uptake, and photosynthetic efficiency, thus increasing plant performance (Yasin et al., 2025).

Rice is a main staple food crop grown globally, and Cd and MPs toxicity are serious challenges for rice growth and productivity (Wang et al., 2025). Thus, it is essential to develop measures to prevent the entry of Cd and MPs into rice fields to improve crop productivity and decrease health risks. Recently, the role of biochar in mitigating Cd and MPs has been well studied; nevertheless, the role of nano-zinc modified biochar in mitigating Cd and MPs has not been studied yet. Therefore, objectives of current study was as follow; 1) elucidating the impacts of MPs on Cd uptake, and accumulation 2) to measure the toxic impacts of co-exposure of Cd and MPs on plant function and soil properties, 3) decipher the role of NZMB on plant physiological functioning, antioxidant defense, gene expression, and Cd uptake in rice grown in Cd and MPs polluted soil.

## Materials and methods

### Biochar preparation

The rice straw was collected, washed, and oven dried (100 °C) for 24 hours, then they were submitted to pyrolysis (500 °C) for 6 hours to prepare the biochar. The zinc oxide nano-particles (ZnO-NPs) were used to produce nano-Zn modified biochar. Zinc NPs precipitation on the biochar surface was done by dissolving the ZnO-NPs (25%) and biochar (75%) in water. Then, the mixture was agitated for 12 hours at 25 °C under a N_2_ atmosphere for minimizing the oxidation. The sample was vacuum-dried (60 °C, 24 hours), and subsequently washed to remove residual impurities. Later, NZMB was subjected to scanning electron microscopy, transmission electron microscopy, and energy dispersive X-ray spectroscopy to determine the different properties of NZMB. Furthermore, a sub-sample of NZMB was taken and subjected to determine the different properties by using the standard procedures. The prepared NZMB had a higher pH of 9.98 and further information about NZMB elemental analysis, FTIR, SEM, and TEM analyses is given in the results section.

### Pot experiment

The experiment was performed at Guangxi Hydraulic Research Institute, Nanning, China from May 2024 to July 2024. The experiment site has a sub-tropical monsoon climate with warm summers and cold winters. The collected soil was first air-dried and then sieved using a 2 mm mesh to eliminate any large debris or particles. The soil was treated with CdCl_2_ salt to get a Cd concentration of 20 mg kg^-1^. Then, Cd-treated soil was stabilized for two months in dark conditions by maintaining 70% field capacity. Then microplastics (1%) were added and soil was stabilized for one week. Thereafter, the soil was taken out of the pots, NZMB was added and allowed to stabilize for one week. After that, pots were filled with water and five seedlings of rice (Zhongjiazao 17) were transplanted in each pot and water was routinely applied and 2–3 cm water level was maintained in each pot. The study had following treatments: control, Cd contaminated soil (20 mg kg^-1^), MPs contaminated soil (1%), Cd + MPs contaminated soil, NZMB (2%), Cd contaminated soil (20 mg kg^-1^) + NZMB (2%), MPs contaminated soil (1%) + NZMB (2%) and Cd + MPs contaminated soil + NZMB (2%). The rate of Cd was selected from previous study (Ahmad et al., 2024), while MPs rate (1%) was also selected from earlier studies of Qi et al. (2018) and Yang and Gao (2022). Additionally, biochar was added at the rate of 2%, and this rate has shown tremendous results in mitigating Cd and MPs toxicity (Xu et al., 2024; Yang et al., 2024). The polyvinyl chloride MPs with a size of 50 µm were used in the study, and they were purchased from Dongguan Siyezi Plastic Co. limited, China. Further information about MPs’ morphology and FTIR analysis is given in the results section.

### Photosynthetic pigments

The fully expanded fresh rice leaves (0.5 g) were homogenized by using 3% acetone (v/v) and placed in dark conditions for 24 hours. Then extract subjected for centrifugation for 10 minutes under 8,000 rpm. Thereafter, chl-a, chl-b, and carotenoids concentration was estimated after reading absorbance at 663 nm, 645 nm, and 479 nm (Lichtenthaler, 1987).

### Oxidative markers, antioxidants activity and osmolytes

The freshly collected rice leaves were taken to measure the different oxidative stress markers. For electrolyte leakage (EL), the freshly collected leaves were turned into 5 mm slices and then soaked in water. Then samples were heated (32 °C) for two hours and first electrical conductivity (EC1) was noted. These samples again incubated (121 °C) for 24 h and EC (EC_2_) was determined, and EL measured as: EL% = (EC_1_/EC_2_) × 100. To measure malondialdehyde (MDA) level, 0.5 g of leaf was chopped in liquid nitrogen at 6.5 pH combined with phosphate buffer to measure the MDA level. Then this extract was centrifuged and mixed with 0.1% TCA and 0.5% TBA, and absorbance was measured at 532 and 600 nm to estimate MDA contents. To estimate the hydrogen peroxide (H_2_O_2_) level, 0.5 g of leaves was taken and crushed by using the 1 mL TCA (0.1%) solution. Then this mixture was centrifuged (8000 rpm) for 15 minutes. Later, mixture comprising of 0.5 mL of PPB and supernatant and 1 mL of potassium iodide was made and reading done at 600 nm to determine H_2_O_2_ concentration. For measuring antioxidant activities 0.5 g leaf samples were collected and crushed using ice and 10 mL potassium phosphate buffer (50 mM with pH 7.8). Then this solution was centrifuged (10000 rpm) for 30 minutes at 4 °C, which was collected to measure activity of different antioxidants. The activities of APX and CAT was measured through protocols of Nakano and Asada (1981) and Aebi (1984), while the POD and SOD was measured by protocols of Zhang (1992). To measure proline 0.5 g of leaves were ground in 3% sulfosalicylic acid solution, and proline concentration was measured by reading absorbance at 520 nm (Bates et al., 1973). Total soluble protein concentration was estimated by crushing 0.5 g fresh leaves in 5 mL PPB. Then, the mixture was centrifuged for 20 minutes, and later it was added with Bradford mixture and absorbance read at 595 nm (Bradford, 1976). The concentration of free amino acids was measured by standard methods of Hamilton and Vanslyke (1943). For this, 1 mL of extract was collected, and it was added with 1 mL pyridine (10%) and ninhydrin (2%) and heated for 30 minutes. Later, the absorbance was taken at 570 nm.

### Measurement of growth and yield traits and cadmium contents

The root and shoots length was measured with a measuring tape, and they were weighed to measure fresh weight. Then samples were dried for determining dry weight. Grains were separated to measure the grain yield, while plants were weighed to estimate biomass yield. The rice roots and shoots were taken, dried, and digested by using two acids (HCl and HNO_3_, 1:2). The digested samples were filtered to remove the impurities and Cd contents was measure by atomic absorption spectrophotometry.

### Measurement of soil properties

Soil samples were collected and debris was removed and pH was determined with pH meter. The nitrogen concentration was measured with the Kjeldahl procedure, while the P and potassium concentrations were measured by using flame-photometer and spectrophotometer methods. Sulfuric acid external heating technique was used for measuring soil organic carbon.

### RNA extraction and qRT-PCR analysis

Leaf tissue samples were collected, and total RNA was isolated with a standard RNA extraction kit. RNA quality and concentration were subsequently verified with a NanoDrop spectrophotometer. Subsequently, cDNA synthesis was performed from 1 µg of RNA with the FastKing gDNA Dispelling RT SuperMix kit for downstream quantitative real-time PCR (qPCR). Additionally, gene expression analysis was carried out via qRT-PCR using the SuperReal PreMix Plus (SYBR Green) kit, with relative quantification determined by the method of Livak and Schmittgen (Livak and Schmittgen, 2001). All analyses were performed in three biological replicates to enhance reliability, with OsActin employed as the reference gene, and the qRT-PCR primer sequences are presented in **Supplementary Table 1**.

### Statistical analysis

The experiment was performed in completely randomized design with three biological replications. The analysis of variance (ANOVA) was performed on the collected data to determine the impact of different treatments by using Statistix 8.1^®^ software. Treatments were compared with honest significant difference (HSD: *p<0.05)* to identify the difference among means. The relationship between different treatments was compared with Principal component analysis and correlation matrix by using R-Studio.

## Results

### Characterization of nano-zinc modified biochar and microplastics

**Figure 1:**
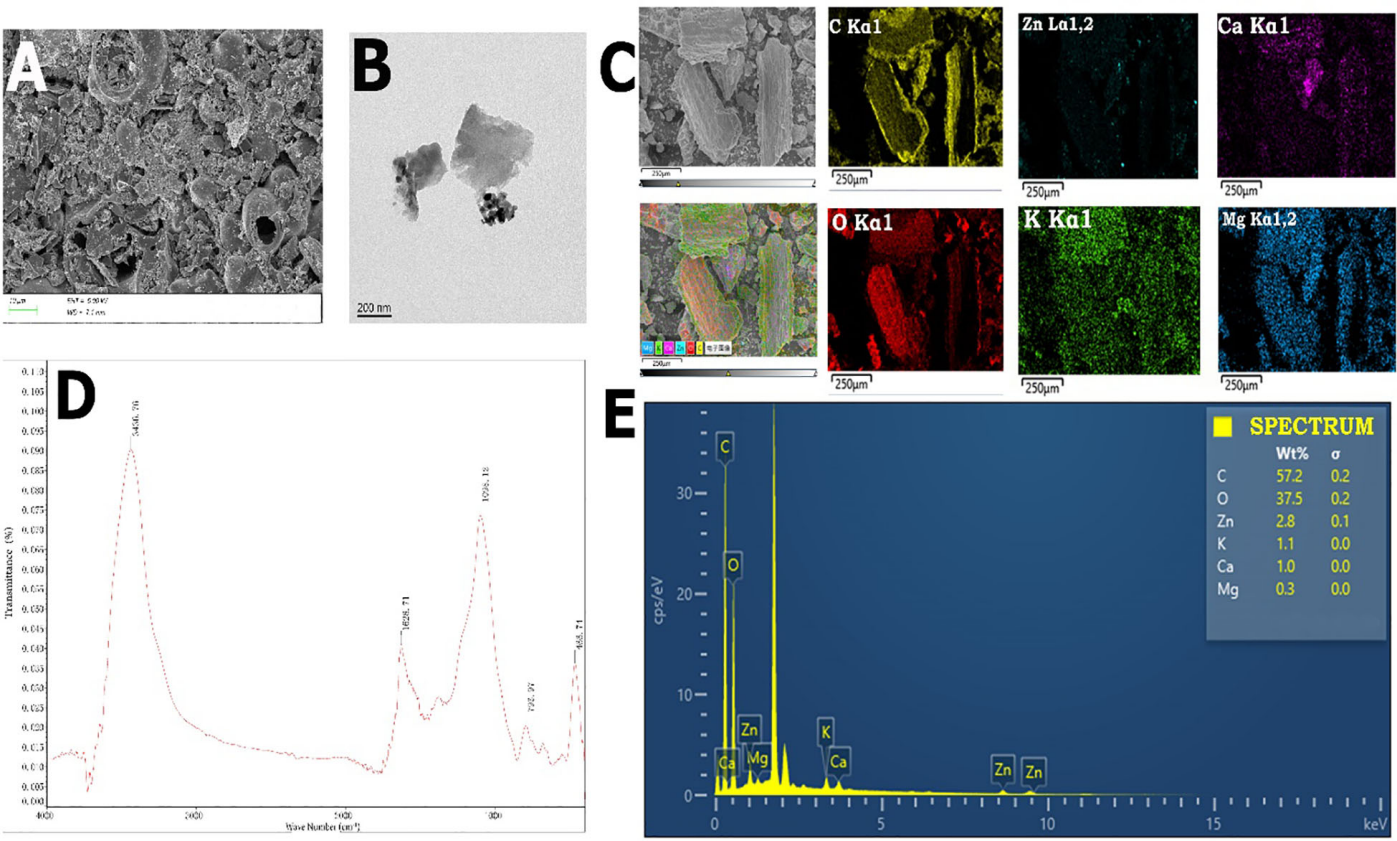
SEM **(A)**, TEM **(B)**, FTIR spectra of biochar **(C)**, elements mapping **(D)** and EDS dispersion of biochar **(E)**.

Surface morphology and characteristics of biochar play a crucial in remediating polluted soils. The prepared NZMB was subjected to FTIR analysis, and it showed peaks at 3436.74, 1628.71, 1098.12, 793.97, and 466.74 which indicates that NZMB contained O-H, C=O, C=C, C-O and C-H groups (**Figure 1**). Biochar was also analyzed by SEM and TEM analysis, indicating that the NZMB has a porous structure. Moreover, EDS analysis was also performed which shows that NZMB had 57.2% carbon, 37.5% oxygen, 2.8% zinc, 1.1% potassium, 1% calcium and 0.3% magnesium (**Figure 1**). The FTIR analysis of MPs showed peaks at different transmittance. The maximum peak was observed at 3429.45, and 2969.10 cm^-1^; following this other peaks were noted at 1629.07, 1430.39, 1095.66 and 421.51 cm^-1^. The peaks observed at 3429.45, and 2969.10 cm^-1^ corresponded to the presence of O-H stretching and C-H stretching; the peaks 1629.07, and 1095.66 cm^-1^ were linked to C=C stretching and C-O stretching. Additionally, peak reported at 421.51 cm^-1^ associated with presence of C-H bending in MPs (**Supplementary Figure 1**). SEM analysis showed that MPs had sharp edges with an irregular surface (**Supplementary Figure 1**).

### Growth and yield traits

**Figure 2:**
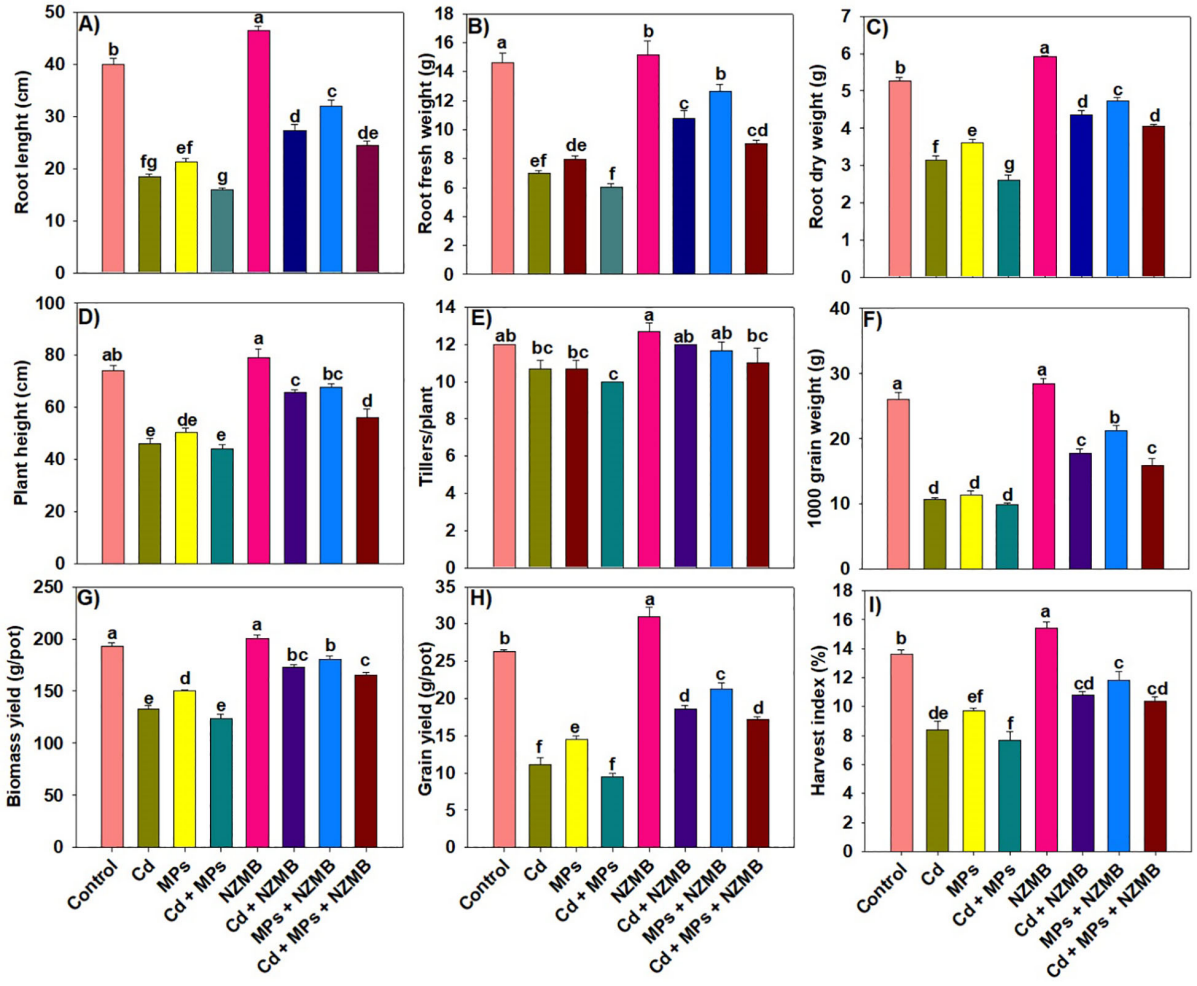
Effects of nano-zinc modified biochar on growth **(a-d)** and yield traits **(e-i)** of rice grown in Cd and MPs contaminated soil. Different letters on bars indicating significance among treatments according to HSD test at *p ≤ 0.05*, while data given in columns is mean of three replications with ± SD. Cd, cadmium; MPs, microplastics; NZMB, nano-zinc modified biochar.

Cadmium and MPs alone, as well as their co-exposure, significantly decreased rice growth, biomass, and grain yields (**Figure 2**). However, NZMB offset the negative impacts of both Cd and MPs, substantially enhancing rice growth and productivity. Cadmium and MPs significantly decreased root growth and biomass productivity; however, maximum reduction was observed with the combination Cd and MPs (**Figure 2**). The supplementation of NZMB increased root length, root fresh and dry biomass by 54%, 50% and 55% respectively, under Cd and MPs co-contaminated soil (**Figure 2**). The application of NZMB also significantly enhanced root growth and biomass under Cd and MPs-contaminated soil and soil without Cd and MPs contamination (**Figure 2**).

Cadmium and MPs stress significantly decreased the plant height and tiller production in rice plants. The results presented a decrease of 68.81%, and 20% in plant height and tillers under combined Cd + MPs stress compared to control (**Figure 2**). Nano zinc modified biochar significantly enhanced plant height and tillers under alone Cd (49.25% and 20%), and MPs stress (53.80% and 16.70%) and combined Cd + MPs stress (27.27% and 10%) conditions (**Figure 2**). Cadmium and MPs caused a significant decrease in rice grain weight, grain and biomass yields; however, maximum reduction was witnessed under combined Cd and MPs stress conditions. Biochar mitigated the decrease and enhanced the grain weight, grain, and biomass yields 60.97%, 33.68% and 81.41% respectively. Further, NZMB enhanced grain weight, grain, and biomass yields 66.07%, 30.14% and 66.60% respectively under alone Cd stress, while NZMB enhanced grain weight, grain, and biomass yields 87.54%, 20.44% and 46.32% respectively under alone MPs stress (**Figure 2**).

### Chlorophyll and leaf water contents

**Figure 3:**
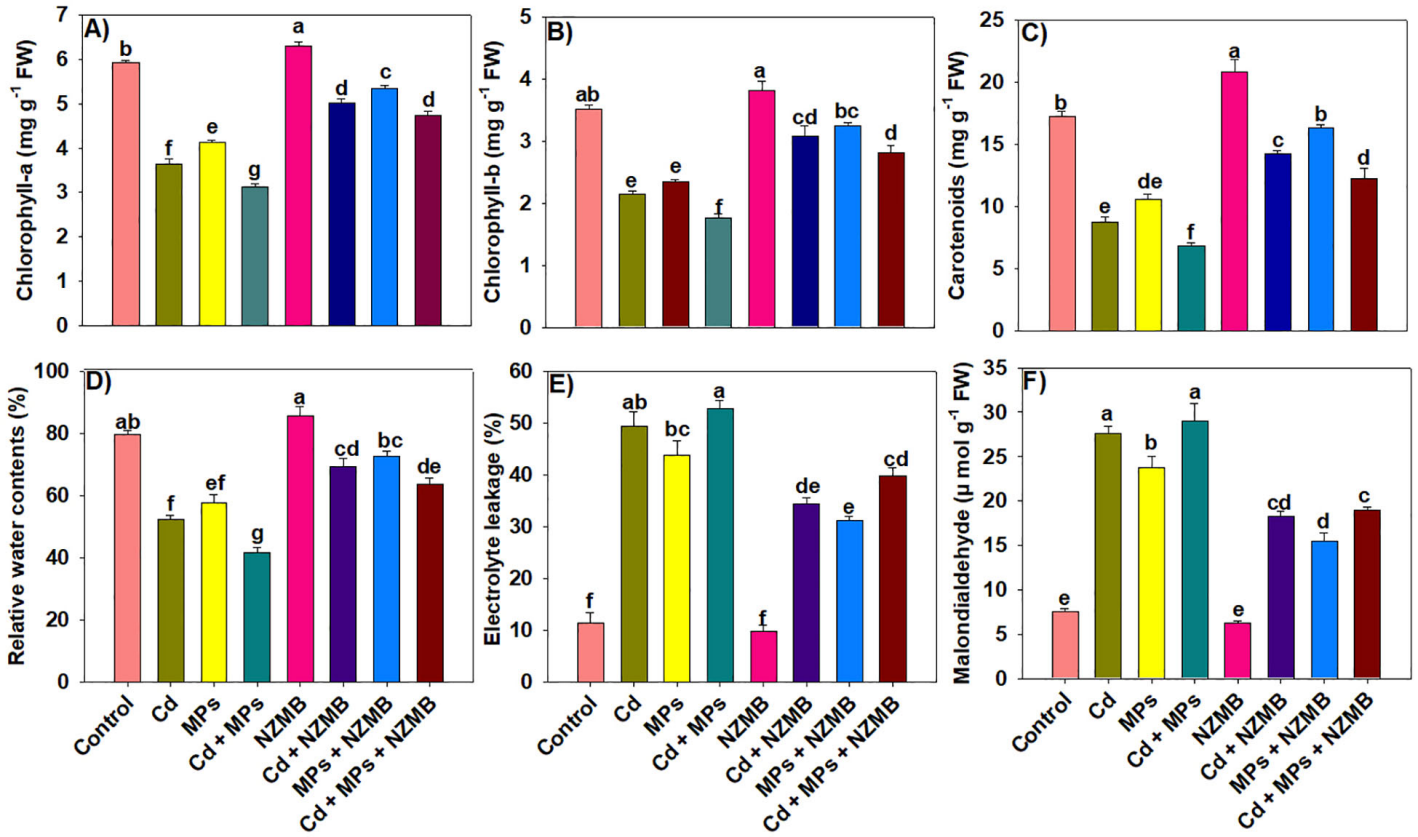
Effects of nano-zinc modified biochar on photosynthetic pigments **(a-c)**, leaf water contents **(d)** and oxidative markers **(e, f)** of rice grown in Cd and MPs contaminated soil. Different letters on bars indicating significance among treatments according to HSD test at *p ≤ 0.05*, while data given in columns is mean of three replications with ± SD. Cd, cadmium; MPs, microplastics; NZMB, nano-zinc modified biochar.

The amount of chlorophyll content is an indication of photosynthetic efficiency. Here, Cd and MPs significantly decreased the Chl-a, Chl-b, and carotenoid contents in rice leaves. This reduction was more pronounced under combined Cd and MPs contaminated soil (**Figure 3**). Nano zinc modified biochar significantly enhanced the chlorophyll and carotenoids synthesis under the sole Cd and MPs and their co-exposure (**Figure 3**). Overall, the maximum chlorophyll and carotenoids were noticed in control and minimum was found in Cd and MPs co-contaminated soil without applying NZMB (**Figure 3**). Cadmium, MPs, and their combination significantly decreased the leaf water contents. The results indicated a reduction of 52.24%, 38.14% and 91.19% respectively in leaf RWC under alone Cd, MP, and combined Cd and MPs stress conditions (**Figure 3**).

### Oxidative stress markers, osmo-protectants and antioxidants activities

Cadmium, MPs, and their co-exposure significantly enhanced the EL, MDA and H_2_O_2_ production in rice leaves, signifying the undesirable impacts of Cd and MPs on rice growth (**Figure 3**). The maximum increase in EL, MDA, and H_2_O_2_ was observed with combined Cd and MPs. Nano zinc modified biochar significantly decreased EL, MDA, and H_2_O_2_ by 32.87%, 53% and 41.02% under combined Cd and MPs stress conditions (**Figure 3**, **Table 1**). The results depicted contrasting impacts of Cd and MPs on osmo-protectants synthesis. The synthesis of proline was increased under Cd and MPs stress, while SP and FAA decreased in response to Cd and MPs. The application of NZMB significantly enhanced the proline, TSP and FAA production under the sole Cd, MPs, and their co-exposure of Cd + MPs (**Table 1**).

**Table 1:** Effects of nano-zinc modified biochar on hydrogen peroxide, and osmolytes synthesis of rice grown in Cd and MPs contaminated soil.

Treatments	Hydrogen peroxide (µmol g^-1^ FW)	Proline (mgg^-1^ FW)	Total soluble proteins (mg g^-1^ FW)	Free amino acids (mg g^-1^ FW)
Control	14.75f±1.35	1.12f±0.057	16.42ab±1.54	10.57b±0.33
Cd	57.42ab±1.66	2.23d±0.040	10.82de±0.99	5.79fg±0.14
MPs	53.74b±1.82	1.92e±0.058	12.21cd±0.86	6.71ef±0.14
Cd + MPs	64.63a±3.37	2.73b±0.015	8.91e±0.09	5.55g±0.18
NZMB	11.57f±1.29	0.94f±0.045	18.71a±0.40	12.46a±0.63
Cd + NZMB	35.22d±2.20	3.63a±0.090	15.11b±0.42	8.49cd±0.19
MPs + NZMB	27.59e±1.70	3.22b±0.120	16.00b±0.048	9.49c±0.23
Cd + MPs + NZMB	45.83c±2.93	3.81a±0.070	14.25bc±0.27	7.65de±0.07

The data given in columns is mean of three replications with ± SD and different letters indicating the significance at *p ≤ 0.05* with HSD test. Cd, cadmium; MPs, microplastics; NZMB, nano-zinc modified biochar.

Antioxidant activity was significantly impacted by Cd and MPs stress and NZMB application.

**Figure 4:**
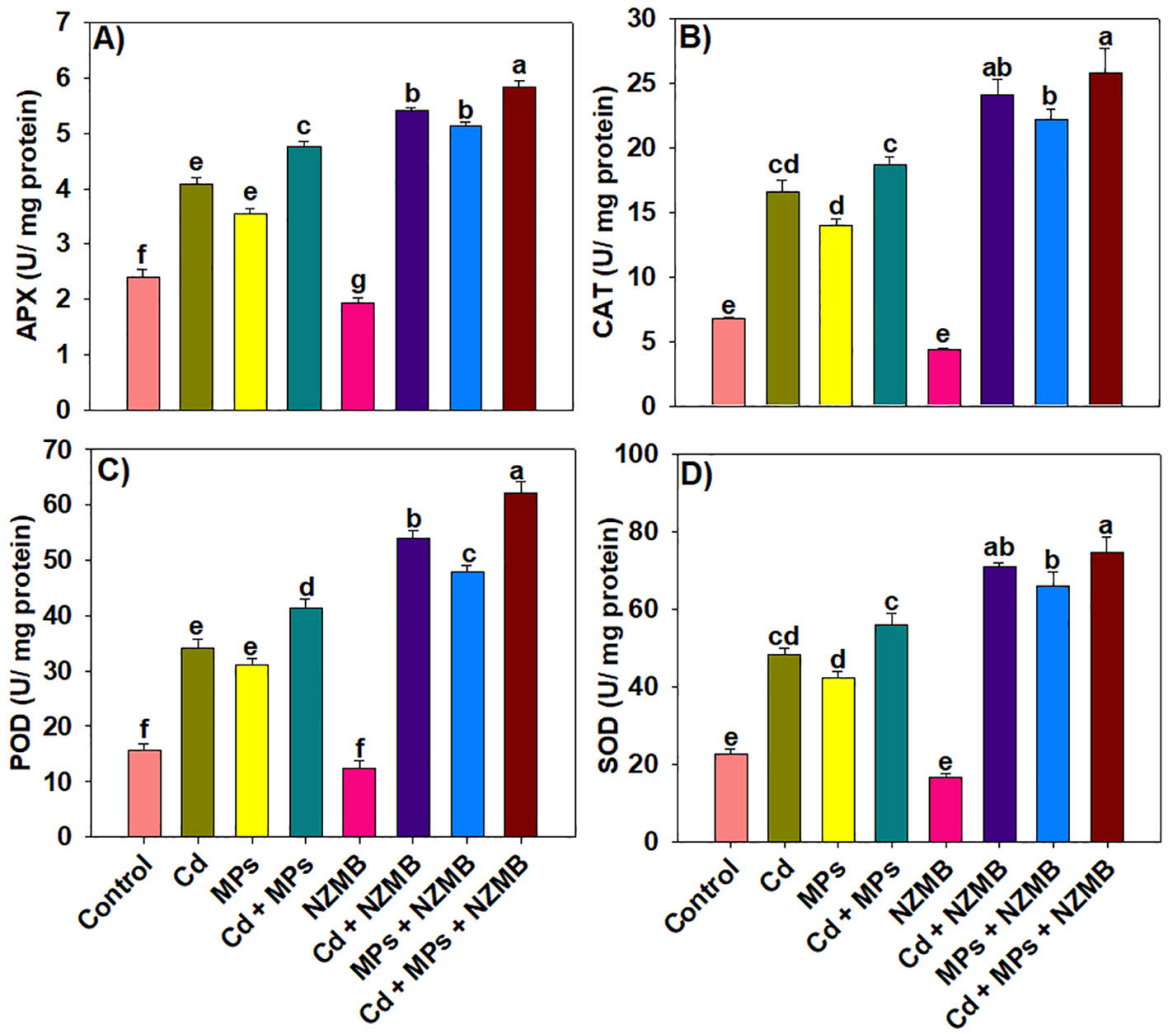
Effects of nano-zinc modified biochar on antioxidant activity **(a-d)** of rice grown in Cd and MPs contaminated soil. Different letters on bars indicating significance among treatments according to HSD test at *p ≤ 0.05*, while data given in columns is mean of three replications with ± SD. Cd, cadmium; MPs, microplastics; NZMB, nano-zinc modified biochar.

The lowest antioxidants were observed in Cd and MPs-free soil with NZMB application, indicating that these plants faced a stress free environment (**Figure 4**). The activity of antioxidants increased under Cd and MPs alone, and it was further increased under combined Cd and MPs. Notably, NZMB enhanced APX, CAT, POD, and SOD activity by 22.47%, 37.88%, 49.95% and 32.85% respectively, under Cd and MPs co-contaminated soil (**Figure 4**).

### Expression patterns of antioxidants, proline synthesis, sucrose transporter and cadmium uptake genes

**Figure 5:**
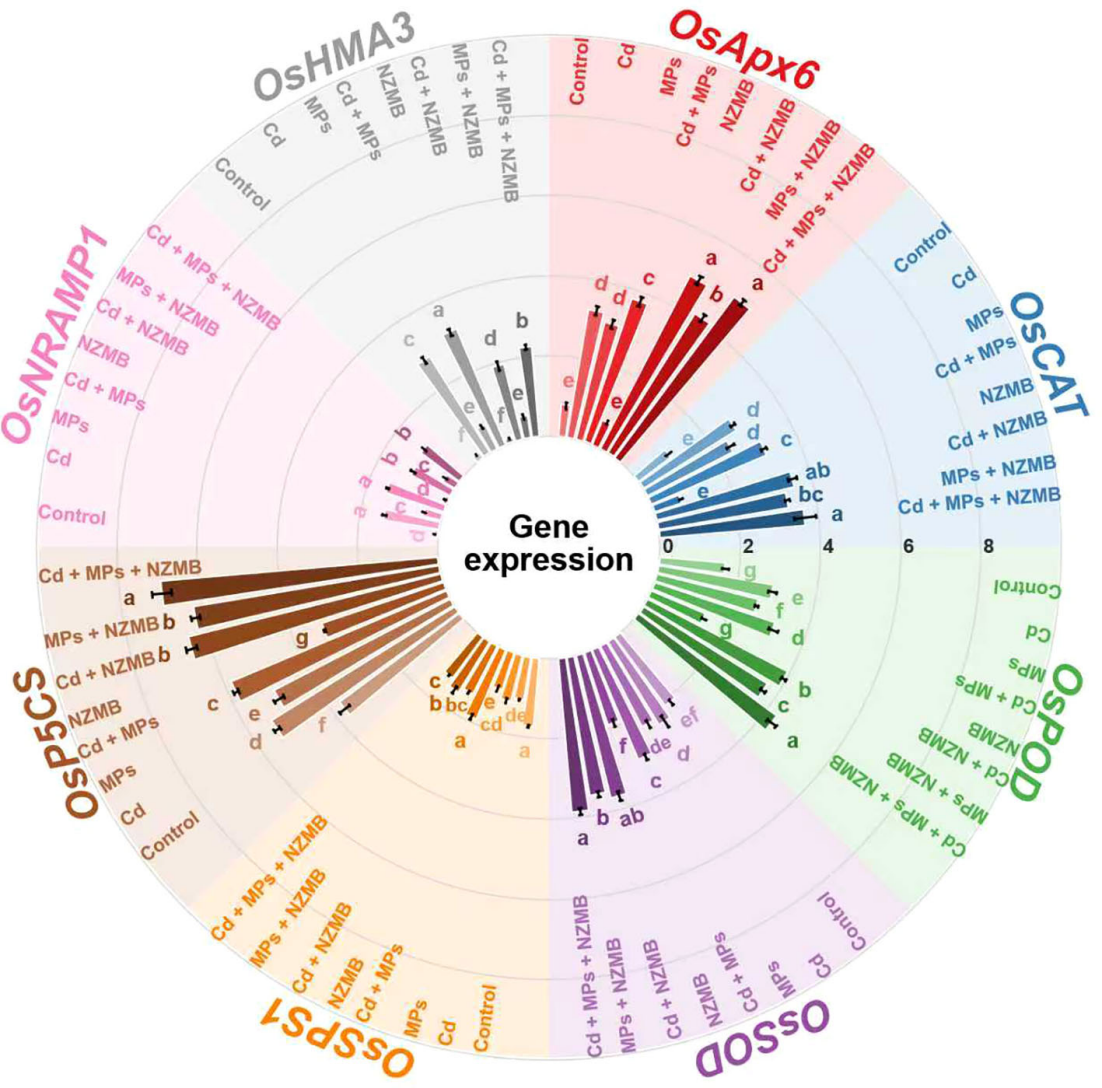
Effects of nano-zinc modified biochar on expression pattern of antioxidants, proline synthesis, sucrose transporter and cadmium uptake genes of rice grown in Cd and MPs contaminated soil. Different letters on bars indicating significance among treatments according to HSD test at *p ≤ 0.05*, while data given in columns is mean of three replications with ± SD. Cd, cadmium; MPs, microplastics; NZMB, nano-zinc modified biochar.

The expression level of antioxidant genes was significantly low in control conditions, and NZMB application to Cd and MPs further decreased the expression of antioxidant genes (**Figure 5**). The expression pattern of *OsAPx6*, *OsCAT*, *OsPOD*, and *OsSOD* was significantly increased under Cd and MPs and their co-exposure conditions. Biochar resulted in a notable increase in expression of antioxidant genes under Cd and MPs, indicating that NZMB mitigates the Cd and MPs toxicity in rice (**Figure 5**). The findings showed that expression of the sucrose synthesis gene *OsSPS1* was decreased under Cd and MPs stress; however, NZMB significantly enhanced expression of *OsSPS1* (**Figure 5**). The expression of the proline synthesis gene *OsP5CS* was considerably enhanced under Cd and MPs stress, indicating that plants increased proline synthesis to counter the Cd and MPs toxicity. Biochar also enhanced the expression pattern of *OsP5CS*, which increased proline synthesis and helped plants in mitigating Cd and MPs stress (**Figure 5**). Cadmium uptake genes (*OsNRAMP1* and *OsHMA3*) expression was enhanced under Cd + MPs stress; however, the maximum increase was seen under combined Cd and MPs stress conditions (**Figure 5**). Biochar significantly decreased the expression of *OsNRAMP1* and *OsHMA3* under Cd and MPs and combined Cd and MPs conditions (**Figure 5**). Biochar decreased the expression pattern of *OsNRAMP1* and *OsHMA3* by 27.73% and 38.93% under Cd and MPs, which helped in decreasing Cd uptake (**Figure 5**).

### Tissue cadmium concentration and soil properties

**Figure 6:**
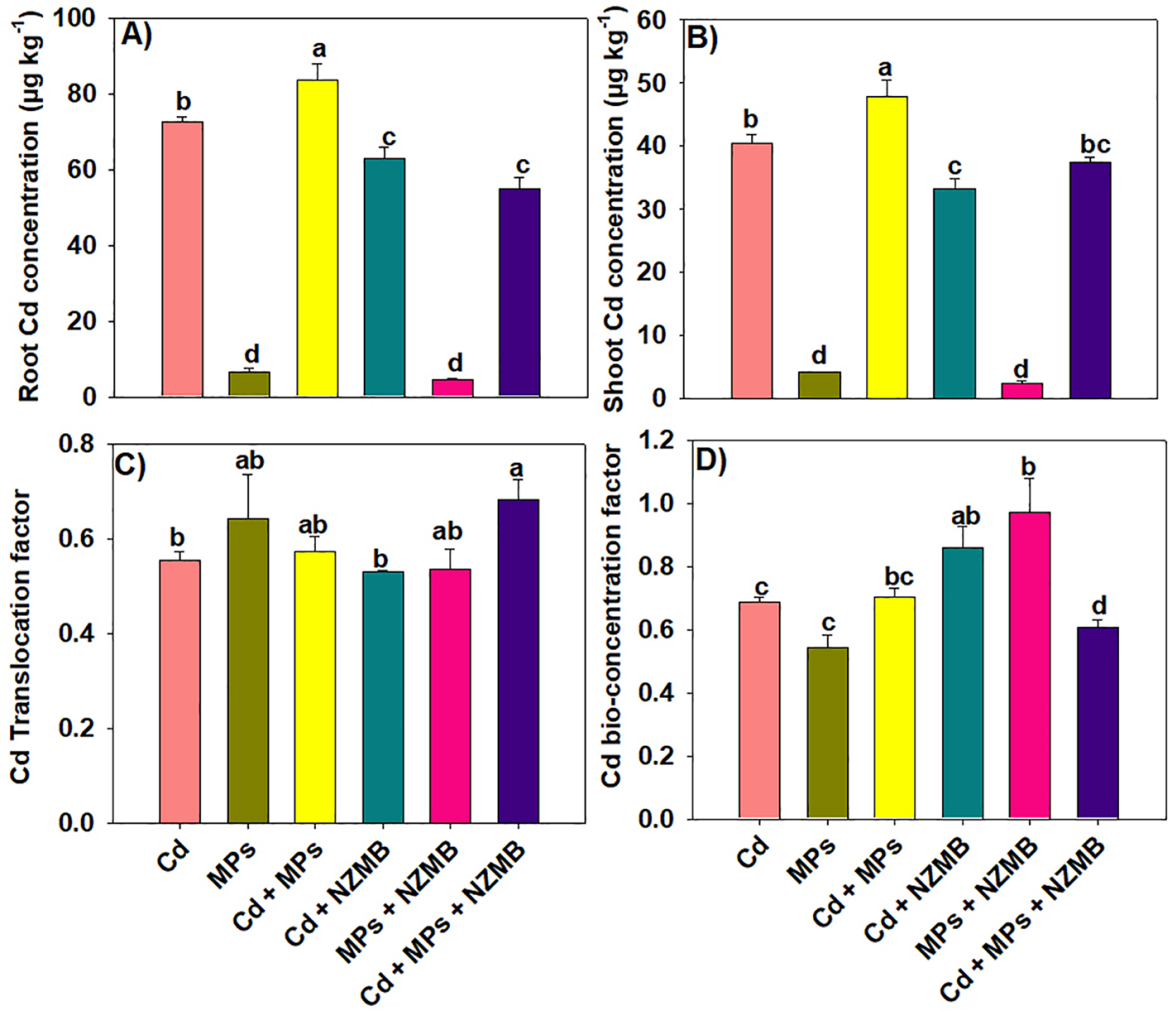
Effects of nano-zinc modified biochar on root and shoot Cd concentration **(a, b)** and Cd translocation factor and bio-concentration factor **(c, d)** of rice grown in Cd and MPs contaminated soil. Different letters on bars indicating significance among treatments according to HSD test at *p ≤ 0.05*, while data given in columns is mean of three replications with ± SD. Cd, cadmium; MPs, microplastics; NZMB, nano-zinc modified biochar.

The results indicated that MPs presence in growing medium boosted Cd uptake and accumulation in rice (**Figure 6**). The maximum Cd contents in the root and shoots were observed under combined Cd and MPs contaminated soil. Biochar caused a marked reduction in Cd accumulation under alone Cd and MPs and co-contamination of Cd and MPs (**Figure 6**). Different treatments significantly impacted the soil Cd availability. Microplastics in Cd-polluted soil significantly enhanced the soil Cd availability compared to Cd alone. Different treatments showed a minor effect on soil pH, and overall Cd and MPs decreased the soil pH, the maximum decrease was observed with their co-exposure (**Table 2**). Soil TN, AP, and AK was significantly decreased with Cd and MPs and their co-exposure. Biochar addition significantly enhanced soil TN, AP, and AK under alone and Cd and MPs contaminated soils and soil co-contaminated with Cd and MPs (**Table 2**). Biochar supply enhanced the soil TN, AP and AK availability by 62.07%, 49.92% and 40.27% respectively, under combined Cd and MPs contaminated soil (**Table 2**). The availability of SOC significantly declined with Cd and MPs; nevertheless, the maximum decrease was observed under combined Cd and MPs. Opposite to NZMB, significantly enhanced SOC by 27.88%, 39.81% and 26.36% respectively in the Cd, MP, and combined Cd and MPs contaminated soil (**Table 2**).

**Table 2:** Effects of nano-zinc modified biochar on soil cadmium concentration, soil pH, nutrients availability and organic carbon in Cd and MPs contaminated soil.

Treatments	Soil DTPA-extractable Cd (µg kg^-1^)	Soil pH	Total nitrogen (g kg^-1^)	Available phosphorous (mg kg^-1^)	Available Potassium (mg kg^-1^)	Soil organic carbon (g kg^-1^)
Control	–	5.66d±0.042	1.35ab±0.024	72.63a±1.33	122.45ab±3.21	21.83b±0.45
Cd	106.10b±2.64	5.50ef±0.016	0.74ef±0.037	37.16de±1.65	77.05fg±4.04	13.27ef±0.89
MPs	12.24e±0.86	5.42e±0.017	0.85e±0.033	40.68d±1.70	86.35ef±4.57	13.84ef±0.50
Cd + MPs	119a±1.88	5.33f±0.037	0.62f±0.053	33.39e±2.26	66.30g±4.58	12.06f±0.47
NZMB	–	6.53a±0.056	1.46a±0.045	76.46a±1.65	132.80a±3.51	24.74a±1.38
Cd + NZMB	73.19b±2.50	6.43ab±0.022	1.12cd±0.021	59.43b±1.49	105.97cd±3.21	16.97cd±0.49
MPs + NZMB	4.86f±0.33	6.35bc±0.026	1.23bc±0.053	65.20b±1.91	113.33bc±4.58	19.35bc±0.64
Cd + MPs + NZMB	90.67d±2.68	6.29c±0.041	1.00d±0.056	50.06c±1.98	93.00de±1.82	15.24de±1.13

The data given in columns is mean of three replications with ± SD and different letters indicating the significance at *p ≤ 0.05* with HSD test. Cd, cadmium; MPs, micro-plastics; NZMB, nano-zinc modified biochar.

### Principal component and correlation analysis

**Figure 7:**
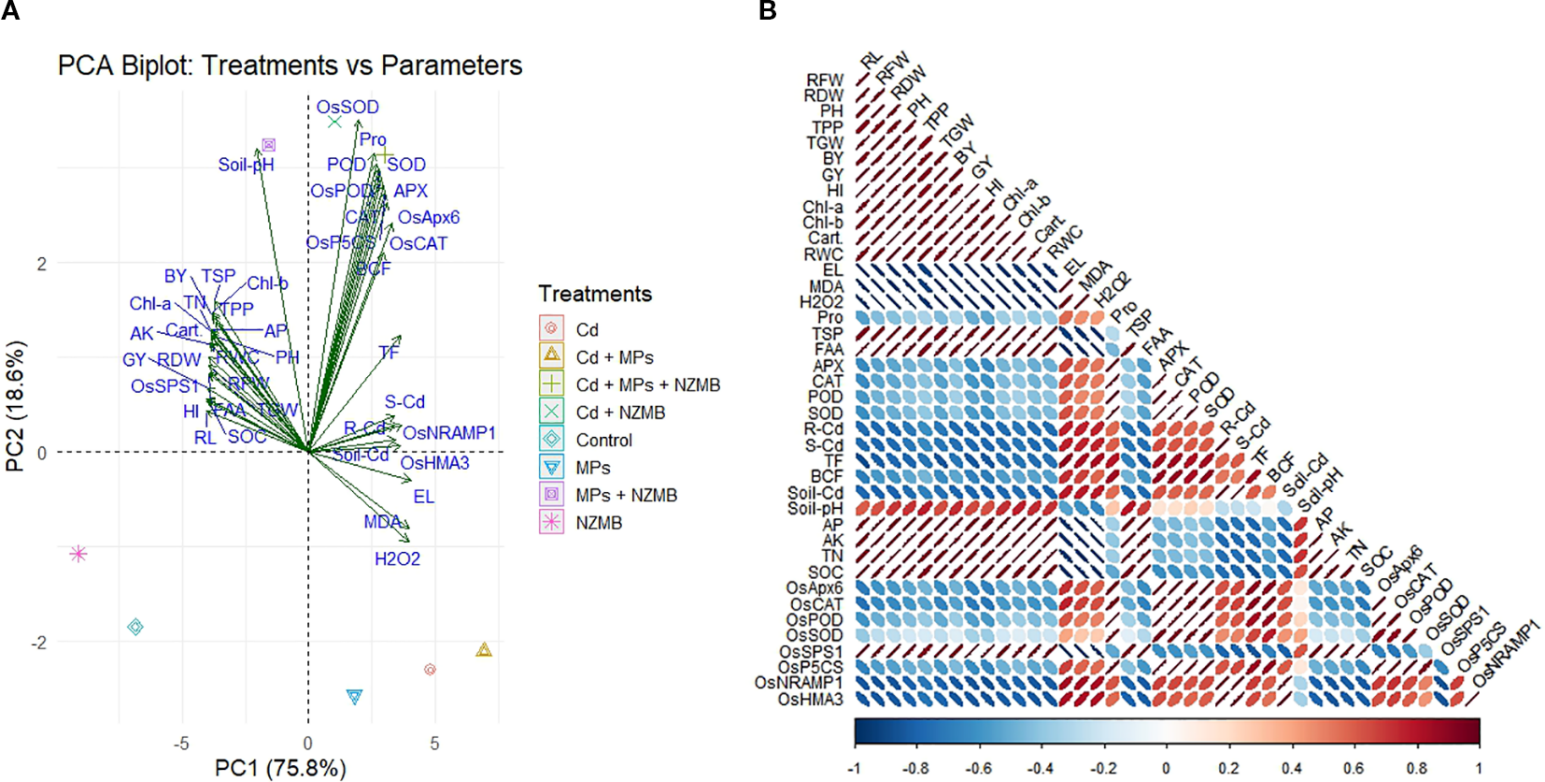
Principal component **(a)** and correlation analysis **(b)** for the effect of different treatment on studied traits. Cd: cadmium, MPs: microplastics, NZMB: nano-zinc modified biochar.

Two components of PCA (PC1 and PC2) had a share of 94.2% with PC1 and PC2 had shares of 78.2% and 16% (**Figure 7a**). The results showed that PC1 distinguished the treatments based on growth and physiological traits, while PC2 distinguished the treatments based on oxidative markers, antioxidants, and Cd uptake and genes expression. The results depicted that Cd and MPs toxicity caused a significant increase in oxidative markers, and application of NZMB showed a substantial decrease in oxidative markers and enhanced rice growth by increasing gene expression, antioxidant activity, and osmolyte production (**Figure 7a**). The growth and yield traits showed a positive association, while, oxidative markers showed a negative linking with RWC, growth and chlorophyll contents. Moreover, antioxidants and osmolytes showed a positive correlation with growth and yield traits, photosynthetic pigments, and RWC. The antioxidants gene expression had positive association with antioxidants. Additionally, SOC and nutrients also had positive association showing the improved soil fertility and reduced Cd availability (**Figure 7b**).

## Discussion

This study was performed to explore the combined effects of Cd and MPs on the growth, physiological functioning, and productivity of rice. The results indicated that combined Cd and MPs decreased rice growth and productivity (**Figure 2**). This decrease in yield was linked with decreased chlorophyll synthesis, water uptake, and increased Cd accumulation, and oxidative damages. Microplastics cause the electrostatic adsorption of Cd and increase its availability. This in turn increases Cd availability and uptake by plants, causing a reduction in photosynthetic efficiency and overall plant growth (Wang et al., 2022). Microplastics also decreased nutrient availability by increasing Cd availability, which has severe competition with nutrients. This decrease in nutrient availability decreases plant growth and yield (Yang et al., 2021). Microplastics absorbed by plant roots decrease the mineral uptake (Erdem et al., 2025) and inhibits microbial activities, thus decreases growth (Fan et al., 2022; Wang et al., 2022). Biochar significantly enhanced the rice yield in Cd and MPs alone and their co-exposure. Biochar used in the current study had a porous structure (**Figure 1**), and rich in carbon (**Figure 1**), which in turn enhanced root growth and subsequently water and nutrients uptake and led to an increase in plant growth (Wei et al., 2023). Biochar also decreased the oxidative stress, hence it enhanced rice growth and yield. Biochar also had high cation exchange capacity (CEC), which increased nutrient availability (**Table 2**), root growth (**Figure 1**), and SOC availability, all of which collectively promote plant growth (Yuan et al., 2023).

**Figure 8:**
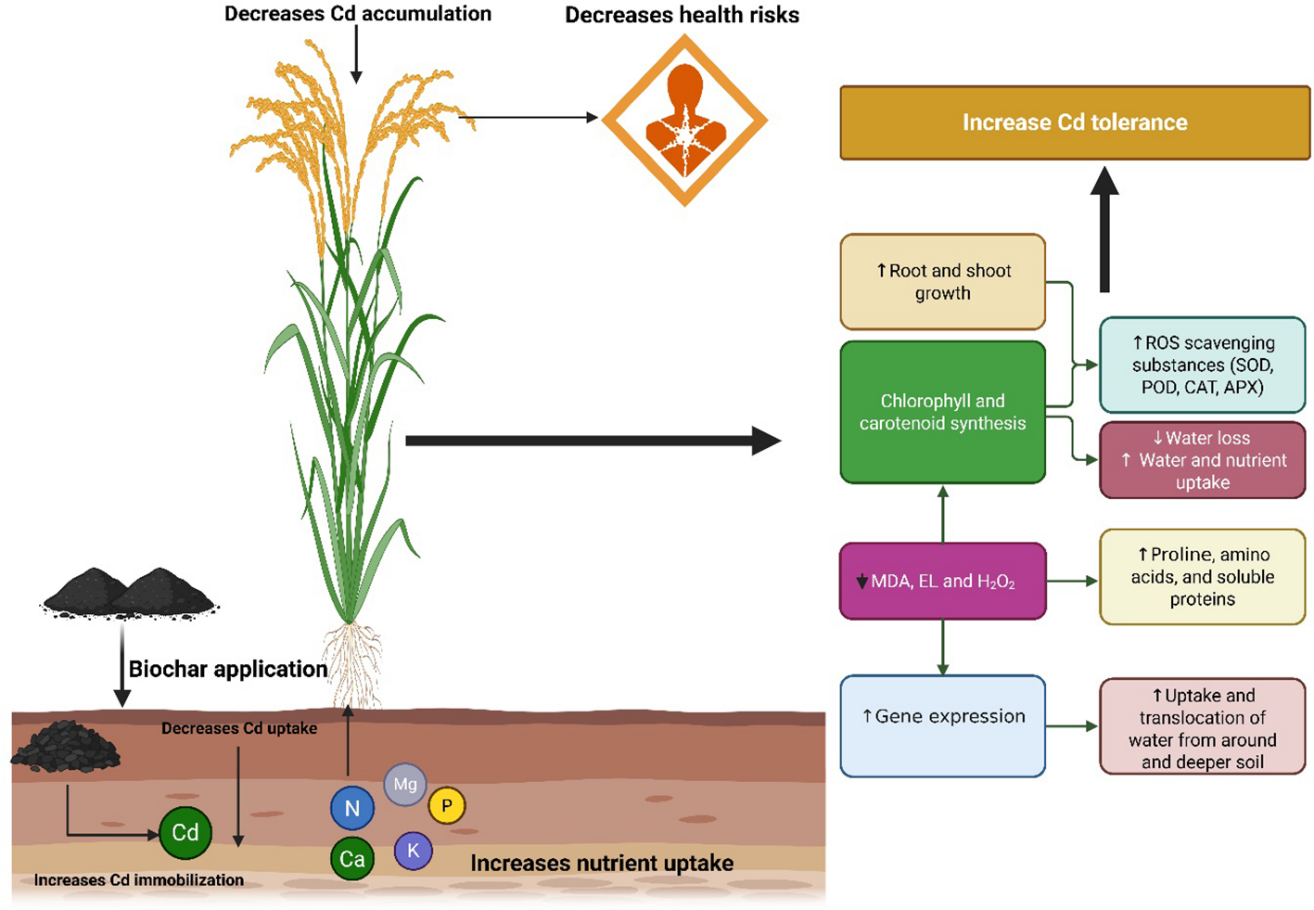
Schematic diagram showing the effects of nano-zinc modified biochar in mitigating the Cd + MPs toxicity in rice.

Cadmium and MPs decreased chlorophyll contents by increasing oxidative stress (**Figure 3**), which might damage chloroplast structure and decrease chlorophyll synthesis. Nevertheless, NZMB significantly enhanced the chlorophyll and carotenoids synthesis (**Figure 8**) by decreasing oxidative stress through an increase in antioxidant activities (**Figure 4**), proline synthesis, and soil nutrient availability (**Table 2**) (Mahamood et al., 2023). Carotenoids are light-absorbing pigments and they work as potent ROS quenchers (Ramel et al., 2012). Cadmium + MPs decreased the carotenoid synthesis, indicating that Cd + MPs augmented ROS production and decreased the ability of carotenoids to scavenge ROS. Leaf RWC was significantly decreased in Cd and MPs stress due to poor root growth (**Figure 3**) and subsequent water uptake. Biochar had a porous structure (**Figure 1**), higher carbon and nutrients (**Figure 1**) and its application increased root growth (**Table 1**), which allowed the plants to uptake more water, thus maintaining better RWC.

Cadmium and MPs toxicity significantly increased the membrane damage (**Figure 3**). In contrast, NZMB caused a marked decrease in oxidative damage by increasing antioxidant activity (**Figure 4**). Nano-Zn modified alleviated the oxidative damage by increasing antioxidant activities, align with early studies (Foong et al., 2022). Biochar increases the antioxidant activity through interactions within the soil-plant system, which in turn provides the defense mechanism to plants, prevents cellular damage, and neutralizes the ROS (Rehman et al., 2019). The increased antioxidant activity driven by NZMB helped the rice plants to counteract Cd and MPs toxicity. This dynamic interaction between NZMB and anti-oxidative responses explores a vital mechanism through NZMB improves plant resilience and yield. Cadmium and MPs significantly decreased the TSP and FAA by decreasing N uptake and its availability. In contrast, Cd and MPs enhanced the proline synthesis. Further, NZMB also enhanced proline synthesis by enhancing *OsP5CS* expression involved in proline synthesis. The increase in proline synthesis mitigate the toxic impacts of abiotic stresses and aid in protecting the plants by stabilizing proteins and membranes (Ma et al., 2024).

Microplastics alter the soil Cd availability, and it largely depends on type, shape and size of MPs (Chen et al., 2023). Here, we observed that MPs augmented Cd uptake owing to the fact MPs work as a vector of Cd. This in turn increased the Cd availability and its accretion in rice plants which is same with early studies reporting same findings (Zhang et al., 2022). Microplastics are vector of Cd and lower soil pH, and they also work as physical abrasion on the surface of plants, thus making them more permeable to Cd. The formation of Cd and MPs complexes (Cd-MPs) also increases the Cd absorption by plant roots. Microplastics have a large surface area (**Figure 2**), which increases Cd absorption on their surface, thereby increasing the Cd availability (Erdem et al., 2025). Microplastic change the soil properties (**Table 2**), which affect the Cd movement and accessibility. Microplastics also release Cd adsorbed by soil particles, which increases Cd availability to plants (Azeem et al., 2021). Nano-Zn biochar significantly decreased Cd uptake and accretion in root and aerial plant parts. Biochar had a significant amount of zinc (**Figure 1**) which has antagonistic relationship with Cd (Farooq et al., 2020), thus it decreased the Cd uptake. Biochar used in the current study had a porous structure (**Figure 1**) with appreciable functional groups (**Figure 1**), which might allow the Cd to adsorb on its surface by complexation and chelation, thus decreasing Cd availability and its accumulation (Severoğlu et al., 2025). Biochar had significant amount of carbon, calcium, Zn, and other beneficial nutrients (**Figure 1**), which increases plant growth (Chen et al., 2024). Additionally, NZMB suppressed the *OsNRAMP1* and *OsHMA3* expression leading to decrease in Cd uptake and accumulation in rice plants. We observed that Cd, MPs, and their co-exposure significantly decreased the soil TN, AP and AK availability (Feng et al., 2022). Microplastics absorb the NH_4_^+^ on the surface, and they also reduce the functioning of genes involved in the N cycle and microbial activities, thus decreasing the soil N availability (Green et al., 2016). Microplastics and Cd also decreases the AP availability by inhibiting microbial activities involved in phosphate-solubilization (Guo et al., 2025). Nano-zinc modified biochar enhanced soil TN, AP, and AK. Biochar increases ion exchangeable sites, ion adsorption, and soil carbon availability, thereby increasing the soil nutrients availability (Silber et al., 2010). This increase in nutrient availability is linked with larger surface and functional groups of NZMB, which improves soil structure, thus increasing the nutrient availability (Hassan et al., 2025). Nano-zinc modified biochar also contained a significant important of nutrients, therefore, its application also directly increased the soil nutrients concentration.

Combined Cd and MPs pollution decreased soil pH. Microplastics release H^+^ ions by changing the soil nitrification process, leading to a decrease in soil pH (Liu et al., 2023). Biochar had alkaline nature (pH: 9.98 pH) and contained alkaline substances (**Figure 1**), therefore, its application enhanced soil pH (Chen et al., 2024). The large surface area of NZMB adsorbs acid substances (H^+^), which reduces their activity, thus preventing the decrease in soil pH (Chen et al., 2024). Furthermore, decomposition of OM present in NZMB also releases alkaline substances (calcium carbonate, calcium hydroxide, and bicarbonate), which also cause an increase in soil pH (Khaliq et al., 2024). We also observed that Cd availability was significantly increased in Cd and MPs-polluted soil, possibly due to a decrease in soil pH (Zhong et al., 2023). Nano-zinc-modified biochar increased soil pH and reduced Cd mobility by forming stable complexes with Cd ions (Chen et al., 2024; Miao et al., 2023).

## Conclusion

Combined cadmium and microplastics stress reduced rice productivity by enhancing cadmium uptake and its accumulation and decreasing soil nutrient availability, soil pH, and impairing plant physiological and biochemical functioning. Nevertheless, nano-Zn modified biochar enhanced the rice production in cadmium and microplastics co-contaminated soil through inhibited cadmium uptake, decreasing cadmium accumulation, and improving plant functioning. The improved yield was also linked with enhanced soil nutrients and carbon availability. These findings suggest that zinc modified biochar could be an important practice to enhance rice yield in cadmium and microplastic co-contaminated soils. This study used only one type and dose of microplastics. Thus, more studies containing different rates and types of microplastics are needed. Further, field studies must be conducted in different environmental conditions to elucidate the role of nano zinc-modified biochar in mitigating combined cadmium and microplastic toxicity. In addition, in-depth omics studies are required to underscore the molecular mechanism mediated by zinc-modified biochar.

## Data Availability

The original contributions presented in the study are included in the article/**Supplementary Material**. Further inquiries can be directed to the corresponding authors.
