# Supplementary material for: Mitigating combined cadmium and microplastics toxicity in rice through nano-zinc modified biochar
Source: Front Plant Sci. 2026 Mar 30;17:1755367. doi: 10.3389/fpls.2026.1755367 (PMC13070825; doi:10.3389/fpls.2026.1755367)
Supplement: Supplementary file 1 [file DataSheet1.docx]

| **Gene** | **Details of primers** |
| --- | --- |
| OsAPx6-F | CCTTCACCTGCGGAACATCT |
| OsAPx6-R | AGCACAGCATCAGTAGGCAG |
| OsCAT-F | GCCACGAAGGACTTGACTGA |
| OsCAT-R | GAGATCCAGATGCCACGGAG |
| OsPOX-F | GAAGGGTTGATGTTGCTGCC |
| OsPOX-R | TCGGCGTTCTTTGATGTCCT |
| OsSOD-F | TACGGGTAGGGCACTGAACA |
| OsSOD-R | CTCCTTTCCGGCAGGATTGT |
| OsSPS1-F | AGATTCGGAGCAAGGTGGTG |
| OsSPS1-R | CTCGTGCGGAGCTTATTTGC |
| OsP5CS-F | TGGAAGATTGGCTTTGGGCA |
| OsP5CS-R | CCCGGAACTTTGGGTTCTCA |
| OsNRAMP1-F | GCCGATCTCAGAAAGGAGCC |
| OsNRAMP1-R | TCCAGGATCGAGGTAAGCCA |
| OsHMA3-F | TGCTGTGAGAACTACGGCTC |
| OsHMA3-R | ATTGCTCAAGGCCATCTGCT |
| Actin-F | CATTGGTGCTGAGCGTTTCC |
| Actin-R | CCCGCAGCTTCCATTCCTAT |

**Table S1:** Details of primers used for gene expression analysis

| 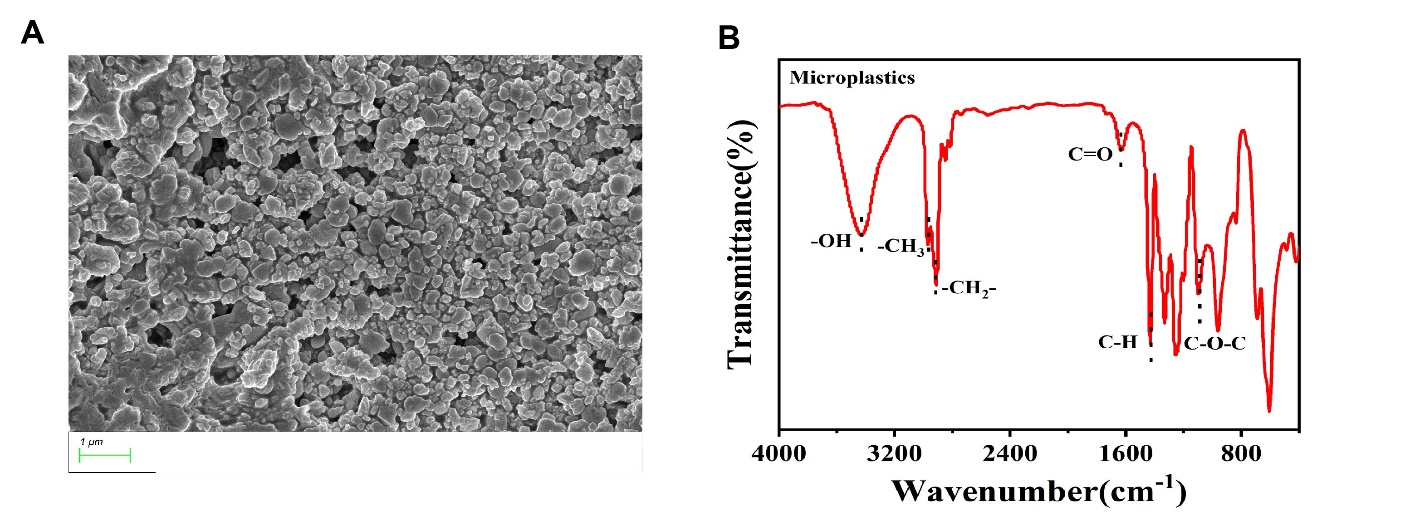 |
| --- |
| **Figure S1:** Scanning electron microscopic (A) and [fourier transform infrared spectroscopy](https://www.google.com/search?q=Fourier+Transform+Infrared+%28FTIR%29+spectroscopy&oq=FTIR+analysis&gs_lcrp=EgZjaHJvbWUqDAgAECMYJxiABBiKBTIMCAAQIxgnGIAEGIoFMgcIARAAGIAEMgcIAhAAGIAEMgcIAxAAGIAEMgcIBBAAGIAEMgcIBRAAGIAEMgcIBhAAGIAEMgcIBxAAGIAEMgcICBAAGIAEMgcICRAAGIAE0gEJNDAzNWowajE1qAIIsAIB8QXOIC1YYMGLEA&sourceid=chrome&ie=UTF-8&mstk=AUtExfDe0zcAxtGFG1f6X5fFvJLI4kwiWo-NLswLAlvEm1orhLb0cr3qWSBRMehoSKA9mi7y_AsGjYLRIZ8vDDZgar2gTzFaLzUtb2c8Wuli_v2PWzfDxNLirQ2JXBeoo0TnNfSSYX4fITqoEYxTfByWUdeEDI4UF04aB4MZQj1aPD2Yrd0&csui=3&ved=2ahUKEwiIyZrz67KSAxXAr1YBHaOsIvwQgK4QegQIARAD) analysis of MPs. |
